# Impact of On-Clopidogrel Platelet Reactivity on Incidence of Peri-Interventional Bleeding in Patients Undergoing Transcatheter Aortic Valve Implantation

**DOI:** 10.3390/jcm11102871

**Published:** 2022-05-19

**Authors:** Alexander Kille, Kilian Franke, Noé Corpataux, Julia Hromek, Christian M. Valina, Franz-Josef Neumann, Dietmar Trenk, Thomas G. Nührenberg, Willibald Hochholzer

**Affiliations:** 1 Department of Cardiology and Angiology II, University Heart Center Freiburg-Bad Krozingen, Medical Center-University of Freiburg, 79189 Bad Krozingen, Germany; kilian.franke@uniklinik-freiburg.de (K.F.); christian.valina@uniklinik-freiburg.de (C.M.V.); franz-josef.neumann@uniklinik-freiburg.de (F.-J.N.); thomas.nuehrenberg@uniklinik-freiburg.de (T.G.N.); 2 Department of Cardiology, Bern University Hospital, University of Bern, 3010 Bern, Switzerland; noe.corpataux@insel.ch; 3 Hegau-Bodensee-Hospital, Studycenter Hegau Bodensee, 78224 Singen, Germany; julia.hromek@glkn.de; 4 Clinical Pharmacology, Department of Cardiology and Angiology II, University Heart Center Freiburg-Bad Krozingen, Medical Center-University of Freiburg, 79189 Bad Krozingen, Germany; dietmar.trenk@uniklinik-freiburg.de; 5 Department of Cardiology and Intensive Care Medicine, Klinikum Wuerzburg Mitte, 97070 Wuerzburg, Germany; willibald.hochholzer@kwm-klinikum.de

**Keywords:** TAVI, TAVR, bleeding, BARC, platelet, aggregometry

## Abstract

Dual anti-platelet therapy (DAPT) with clopidogrel and acetylsalicylic acid (ASA) has previously been recommended after transcatheter aortic valve implantation (TAVI) and is still the standard of care in patients who underwent coronary stent placement within 3 months prior to TAVI. This study sought to evaluate whether on-treatment platelet reactivity is a predictor for the occurrence of bleeding events after TAVI. This study enrolled 484 patients undergoing TAVI from November 2013 until April 2018. Patients were either on long-term DAPT with clopidogrel and ASA or received loading doses of both drugs before TAVI, reflecting the standard of care at the time of the patient’s enrollment. Platelet reactivity was determined by multi-electrode impedance aggregometry before TAVI, at days 1 and 5 thereafter. Peri-interventional bleeding was assessed up to 5 days following TAVI and coded according to BARC-classification. Bleeding events were seen in 199 (41.1%) patients. The most frequent were BARC 2 bleeding cases (24.2%), followed by BARC 1 (6.0%), BARC 3b (5.2%), and BARC 3a (4.5%) cases. Low on-clopidogrel platelet reactivity before TAVI was present in 243 patients, of which 44.4% had a bleeding event. In contrast, the incidence of bleeding was 30.5% in the 95 patients with high on-clopidogrel platelet reactivity. Multivariate logistic regression analysis identified low/normal/high on-clopidogrel platelet reactivity (OR: 0.533; CI: 0.309–0.917; *p* = 0.023) and use of oral anticoagulation (OR: 1.766; CI: 1.209–2.581; *p* = 0.003) as strongest predictors for peri-interventional bleeding events. These findings support current recommendations advocating against the routine use of dual antiplatelet therapy following TAVI.

## 1. Introduction

Despite limited evidence, dual antiplatelet therapy (DAPT) for several months after transcatheter aortic valve implantation (TAVI) was considered standard of care in patients with no indication for oral anticoagulation (OAC) [1,2]. The rationale for DAPT was to prevent valve thrombosis. In the following years, the first cases of hypo-attenuated leaflet thickening were reported [3], with ongoing controversy on its clinical relevance. While this complication was reported to occur rarely in patients with an indication for OAC [4], it remained uncertain if there was a need for clopidogrel on top of OAC after TAVI. Recently, the POPular-TAVI randomized clinical trial demonstrated an excess in bleeding for a combination of OAC or acetylsalicylic acid (ASA) with clopidogrel [5]. Likewise, ESC/EACTS guidelines now recommend lifelong single antiplatelet therapy in patients without an indication for OAC and a lifelong OAC without antiplatelet therapy if there is an indication for OAC after TAVI [6]. In contrast, ACC/AHA guidelines suggest DAPT with ASA and clopidogrel for 3–6 months, followed by lifelong ASA therapy in patients with low bleeding risk after TAVI [7]. Concerning the multiple patients with an indication for DAPT after TAVI, mainly due to recent coronary stent placement, it seems obvious that they might be at an increased risk for bleeding events. Yet, on-clopidogrel platelet reactivity is highly heterogeneous in patients due to large drug response variability [8,9] and other factors [10,11]. While it is known that different levels of on-clopidogrel platelet reactivity are not associated with the development of early hypo-attenuated leaflet thickening [12], there is only a little information [13] regarding the association between peri-interventional bleeding following TAVI and platelet reactivity. Therefore, this study investigated whether the risk of early bleeding after TAVI is associated with levels of on-clopidogrel as well as on-ASA platelet reactivity.

## 2. Materials and Methods

This is a secondary analysis of a study investigating the impact of on-clopidogrel platelet reactivity on the incidence of hypo-attenuated leaflet thickening after TAVI [12]. For this analysis, 484 patients undergoing TAVI from November 2013 until April 2018 were enrolled. They were either on long-term DAPT (>5 days) with clopidogrel (75 mg once daily) and ASA (100 mg once daily) or received a loading dose of 300 or 600 mg of clopidogrel and 400 mg ASA the day before TAVI. Clopidogrel 75 mg and ASA 100 mg daily were continued after the procedure. Patients on other P2Y12-inhibitors such as ticagrelor were not enrolled. OAC was stopped the day before TAVI and started again two days thereafter. In the meantime, low-molecular-weight heparin or unfractionated heparin (goal partial thromboplastin time 60–80 s) were administered.

Arterial blood samples for platelet function testing (in recombinant-hirudin-anticoagulated blood; 6.4 mM r-hirudin/mL), clinical chemistry (in heparin anticoagulated blood), and hematology (in ethylenediaminetetraacetic acid–anticoagulated blood) were drawn at the beginning of the TAVI procedure before heparin was administered, on day 1 and days 5 to 7. Platelet function testing was processed within 30 min after collection using the ADP test and ASPI test by multiple electrode impedance aggregometry (Multiplate analyzer, Roche Diagnostics, Mannheim, Germany), as previously described [14]. Standard hematological testing was performed by a Sysmex XE-2100 (Sysmex, Norderstedt, Germany) and clinical chemistry testing by a Cobas C501 (Roche Diagnostics, Rotkreuz, Switzerland).

The access route for TAVI was transfemoral but transapical access was also allowed for analysis. In all transfemoral cases, Perclose Proglide^TM^ (Abbott, Santa Clara, CA, USA) was used as vascular closure device and for a second 6F sheath FemoSealTM (Terumo, Tokyo, Japan) or Angio-Seal^TM^ (Terumo, Tokyo, Japan).

Bleeding events were categorized by the Bleeding Academic Research Consortium Definition for Bleeding (BARC) [15].

For statistical analysis, cut-off values of <190 AU x min for low and >468 AU x min for high on-clopidogrel platelet reactivity were used, which were identified and validated in previous studies of patients undergoing coronary stenting [16,17]. Continuous variables are reported as medians with interquartile ranges and discrete variables as counts. A Chi-square test was performed to test for differences between discrete variables, for continuous variables the Mann–Whitney U was performed. To test if platelet reactivity or other variables were predictive for bleeding events, univariate and multivariate binary logistic regression models were performed. Odds ratios (OR) are displayed with 95% confidence intervals (CI) and all tests were performed as 2-sided tests with alpha = 0.05. Statistical testing was done with SPSS version 25 (IBM Corporation, Armonk, NY, USA).

## 3. Results

For this analysis, 484 patients were enrolled (Table 1). In these patients, 199 bleeding events, from mild to severe, were recorded. The most common were BARC 2 bleedings (117; 24.5%) followed by BARC 1 (29; 6.0%), BARC 3b (25; 5.2%), and BARC 3a (22; 4.5%) bleedings (see Table 2). By location, most bleeding events occurred at the vascular access sites (88 (44.2%)). Most of them were cutaneous minor BARC 2 bleedings (43) which did not affect the punctured vessel itself. However, if the vessel was affected, it mostly caused a major bleeding event (29 (54.7%) of 53 major bleedings). Gastrointestinal bleedings (7 (12.9%)) were the second most common major bleedings. There were 4 fatal bleedings: A periinterventional rupture of the annulus and severe pulmonary bleeding after TAVI, and two bleedings of unknown cause into the pericardium which led to pericardial tamponade. One of them occurred on day 1 after TAVI and the other on day 5 after the procedure.

Access route for TAVI was transapical in 2 cases and transfemoral in all other cases. For the second 6F sheath, FemoSeal^TM^ was used in 251 cases and Angio-Seal^TM^ in 221 cases. In 10 cases, no other closure devices were used because of procedural issues. There was no difference in bleeding events (97 vs. 96 vs. 4; *p* = 0.571) for the closure devices.

**Figure 1:**
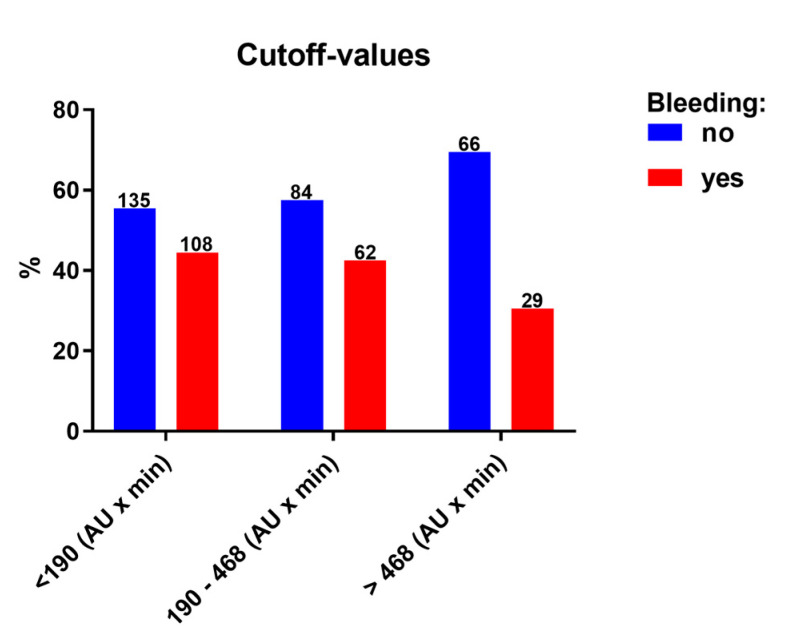
High and low on platelet reactivity: The distribution of patients with and without bleeding complications, based on established cut-off values for low or high on clopidogrel platelet reactivity, is shown.

Low on-clopidogrel platelet reactivity before TAVI was present in 243 patients, of which 44.4% had a bleeding event. In contrast, the incidence of bleeding was 30.5% in the 95 patients with high on-clopidogrel platelet reactivity (Figure 1; Table 3). A total of 146 patients were classified neither as low nor as high on-clopidogrel platelet reactivity; 42.5% of these patients suffered from bleeding. Comparing these 3 groups of platelet reactivity, there was a trend toward a rising incidence of bleeding with lower on-clopidogrel platelet reactivity (*p* = 0.06). There were significantly more bleeding events in patients with low on-clopidogrel platelet reactivity than in the group with high on-clopidogrel platelet reactivity (*p* = 0.019).

**Figure 2:**
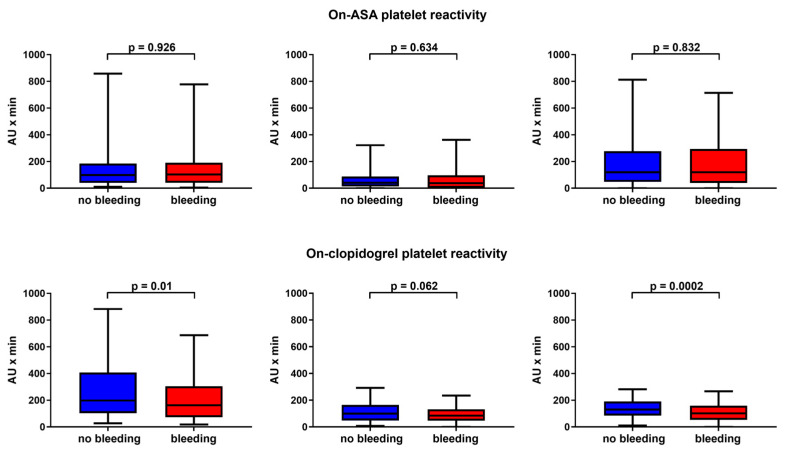
Platelet reactivity: Shown is the platelet reactivity measured by multiple electrode impedance aggregometry before TAVI, on day 1 and day 5. ASA = acetylsalicylic acid.

The baseline characteristics are shown in Table 1. The only statistically significant difference between patients with or without bleeding event was a higher prevalence of OAC in the group with bleeding (33.0% vs. 47.2%; *p* = 0.001). There was no difference if patients were already on long-term therapy with clopidogrel (24.6% vs. 25.1%; *p* = 0.737) or ASA or not (51.2% vs. 49.7%; *p* = 0.749). Loading with clopidogrel was most commonly done with 300 mg p.o. (81.4%). The time between loading with clopidogrel and TAVI was 18.85 [16.42–22.5] hours. A total of 121 patients received a maintenance dose of 75 mg clopidogrel p.o. before TAVI. Median on-clopidogrel platelet reactivity was 179 [96–369] AU x min and median on-ASA platelet reactivity 99 AU x min [41–187] at the beginning of TAVI procedure (see also Supplementary Table S1 for all values). There was a significant difference in on-clopidogrel platelet reactivity between the no bleeding and bleeding group before TAVI (198 [103–408] AU x min vs. 163 [73–305] AU x min; *p* = 0.01) and on day 5 (130 [85–191] AU x min vs. 101 [54–159] AU x min; *p* = 0.0002), while there was only a trend on day 1 (101 [48–166] AU x min vs. 85 [47–132] AU x min *p* = 0.062; Figure 2). Regarding on-ASA platelet reactivity, there was no difference before TAVI (99 [41–184] AU x min vs. 104 [41–191] AU x min; *p* = 0.926), day 1 (40 [14–88] AU x min vs. 38 [3–97] AU x min; *p* = 0.634), and day 5 (120 [48–277] AU x min vs. 120 [40–294] AU x min; *p* = 0.832; Figure 2). There was no difference in on-clopidogrel platelet reactivity before TAVI if patients were grouped by presence or absence of major bleedings (180 AU x min vs. 171 AU x min; *p* = 0.951) or by the indication for OAC (178 AU x min vs. 192 AU x min; *p* = 0.176).

To test if other variables then on-clopidogrel platelet reactivity may contribute to the risk for bleeding, univariate and multivariate logistic regression analyses were performed (Table 4). Initially, known predictors of bleeding were tested in univariate models. On-ASA platelet reactivity (OR: 1.0; CI: 0.999–1.001; *p* = 0.806), age (OR: 0.994; CI: 0.961–1.029; *p* = 0.751), hemoglobin (OR: 1.038; CI: 0.933–1.115; *p* = 0.493), and platelet count (OR: 0.998; CI: 0.995–1.000; *p* = 0.087) were not predictive for bleeding events. On-clopidogrel platelet reactivity stratified by low/normal/high on-clopidogrel platelet reactivity (OR: 0.549; CI: 0.322–0.910; *p* = 0.020) and indication for OAC (OR: 1.837; CI: 1.265–2.666; *p* = 0.001) showed significant test results and were included in multivariate testing as well as platelet count which showed a trend. The multivariate model identified stratified by low/normal/high on-clopidogrel platelet reactivity (OR: 0.533; CI: 0.309–0.917; *p* = 0.023) and indication for OAC (OR: 1.766; CI: 1.209–2.581; *p* = 0.003) as independent predictors for bleeding.

## 4. Discussion

Several randomized studies [5,18,19,20] have changed our understanding of the need for an additional therapy with clopidogrel after the TAVI procedure and led to changes in the current guidelines [6,7]. While the optimal time point of elective coronary intervention with regard to a TAVI procedure is currently evaluated in the TAVI-PCI trial (ClinicalTrials.gov Identifier: NCT04310046) [21], it is common practice to perform coronary intervention shortly prior to TAVI owed to the usually easier coronary access. Thus, there is still a significant proportion of patients needing DAPT after TAVI due to recent coronary intervention before TAVI. Our study shows that there is a higher risk for bleeding after TAVI in patients who have a low on-clopidogrel platelet reactivity or an indication for OAC. Low on-clopidogrel platelet reactivity increased mainly the risk for minor bleedings, which might have less impact on other clinical outcomes such as mortality as compared to major bleeding. The majority of minor bleedings occurred at the inguinal puncture site affecting the subcutaneous tissue but not the vessel itself. If the vessel was the source of bleeding, it usually led to a major bleeding event regardless of whether or not the patients showed an indication for OAC or a low on-clopidogrel platelet reactivity. We did not observe an association between on-ASA platelet reactivity and bleeding events in our study. Yet, all patients were on clopidogrel and ASA, so a potential general effect of ASA therapy on bleeding events cannot be excluded by our study. Indeed, it is known that bleeding events also occurred in patients undergoing TAVI if they are on ASA monotherapy [20,22]. This study was not powered to analyze if the previously described cut-off value for a high bleeding risk in patients on clopidogrel undergoing coronary stenting [17] is also suitable for patients undergoing TAVI. Still, we could observe a statistical trend (*p* = 0.06) for more bleeding events in the group of patients with low on-clopidogrel platelet reactivity (<190 AU x min). Comparing patients with low versus high (>468 AU x min) on-clopidogrel platelet reactivity, there were more bleeding events in the group with low on-clopidogrel platelet reactivity (*p* = 0.019)

This is a secondary analysis of a study investigating the impact of on-clopidogrel platelet reactivity on the incidence of hypo-attenuated leaflet thickening [12]. We showed that the risk of bleeding is enhanced if patients display low on-clopidogrel platelet reactivity and that there is a higher risk for bleeding if they are on OAC, usually due to atrial fibrillation. The results of our study are in line with current guidelines but also add important information for clinicians: patients undergoing TAVI are at high risk for bleeding. Low on-clopidogrel platelet reactivity and an indication for OAC are associated with an increased risk mainly for minor bleedings but not for major bleedings. Even if minor bleedings have usually less clinical impact than withholding treatment for severe aortic stenosis, or a stent thrombosis, which may be triggered by withholding clopidogrel, these data clearly support the current ESC guidelines for avoiding DAPT after TAVI if not indicated otherwise [6]. In addition, minor bleedings impact patients’ well-being, can trigger other major clinical events, and should be reduced by adherence to current guidelines [6].

## 5. Conclusions

On-clopidogrel platelet reactivity and use of oral anticoagulation are predictive for periinterventional bleeding but were not found to be associated with the occurrence of major bleeding events after TAVI.

## Figures and Tables

**Table 1:** Baseline characteristics.

		Total = 484	No Bleeding = 285	Bleeding = 199	*p*-Value
Age (years)		83 [79–86]	83 [79–86]	83 [80–86]	0.938
Female		264 (54.5%)	164 (57.5%)	100 (50.3%)	0.113
Body mass index (kg/m^2^)		25.8 [23.5–28.8]	25.7 [23.1–29.2]	26.1 [23.6–28.4]	0.760
Body mass index (kg/m^2^) > 30		96 (19.8%)	61 (21.4%)	35 (17.6%)	0.300
Hypertension		438 (90.5%)	258 (90.5%)	180 (90.5%)	0.978
Diabetes mellitus type II		136 (28.1%)	79 (27.7%)	57 (28.6%)	0.079
History of smoking		91 (18.8%)	49 (17.2%)	42 (21.1%)	0.717
History of coronary disease		345 (71.3%)	196 (68.8%)	149 (74.9%)	0.144
Previous CABG		46 (9.5%)	26 (9.1%)	20 (10.1%)	0.705
Positive family history for CAD		69 (14.3%)	42 (14.7%)	27 (13.6%)	0.717
History of pAOD		56 (11.6%)	36 (12.6%)	20 (10.1%)	0.382
Creatinine clearance (CKD-EPI; mL/min)		52.0 [38.0–69.0]	52.0 [39.0–69.0]	52.0 [37.1–68.7]	0.812
Platelets (10^3^/µL)		208 [164–255]	215 [170–256]	202 [159–251]	0.104
STS mortality score		4.0 [2.7–5.6]	4.0 [2.7–5.6]	3.9 [2.7–5.9]	0.985
Transapical access route		2 (0.4%)	0 (0%)	2 (1.0%)	0.169
Valve type	Balloon-expandable	318 (65.7%)	193 (67.7%)	125 (62.8%)	0.263
	Self-expandable	166 (34.3%)	92 (32.3%)	74 (37.3%)	
Pacemaker after TAVI		71 (14.7%)	42 (14.75%)	29 (14.7%)	0.665
On long term ASA		245 (50.6%)	146 (51.2%)	99 (49.7%)	0.749
On long term clopidogrel		120 (24.8%)	70 (24.6%)	50 (25.1%)	0.737
Indication for oral anticoagulation		188 (36.8%)	94 (33.0%)	94 (47.2%)	0.001

Shown are the baseline characteristics. ASA = acetylsalicylic acid; CABG = coronary artery bypass graft; CAD = coronary artery disease; pAOD = Peripheral arterial occlusive disease; STS = Society of Thoracic Surgeons; TAVI = transcatheter aortic valve implantation.

**Table 2:** Bleeding events.

	BARC 1	BARC 2	BARC 3a	BARC 3b	BARC 3c	BARC 5a + b	All
Hematoma (any location)	20 (69.0%)	8 (6.8%)	3 (13.6%)	1 (4.0%)	-	-	32 (16.1%)
Central/peripheral venosus access	2 (6.9%)	25 (21.4%)	-	-	-	-	27 (13.6%)
Puncture site inguinal (surface)	1 (3.4%)	43 (36.8%)	1 (4.5%)	2 (8.0%)	-	-	47 (23.6%)
Puncture site inguinal (vessel)	-	12 (10.3%)	9 (40.9%)	20 (80.0%)	-	-	41 (20.6%)
Gastrointestinal	-	2 (1.7%)	7 (31.8%)	-	-	-	9 (4.5%)
Macrohematuria	-	10 (8.5%)	1 (4.5%)	-	-	-	11 (5.5%)
Pericardial tamponade	-	-	-	1 (4.0%)	-	3 (75.0%)	4 (2.0%)
Cerebral	-	-	-	-	2 (100.0%)	-	2 (1.0%)
Epistaxis	4 (13.8%)	6 (5.1%)	1 (4.5%)	-	-	-	11 (5.5%)
PM-pocket	2 (6.9%)	6 (5.1)	-	-	-	-	8 (4.0%)
Pulmonary	-	1 (0.9%)	-	1 (4.0%)	-	1 (25.0%)	3 (1.5%)
Others	-	2 (1.7%)	-	-	-	-	2 (1.0%)
Multiple	-	2 (1.7%)	-	-	-	-	2 (1.0%)
All	29 (14.6%)	117 (58.8%)	22 (11.1%)	25 (12.5%)	2 (1.0%)	4 (2.0%)	199 (100%)

Bleedings events categorized by BARC definition; PM = pacemaker.

**Table 3:** Severity of bleeding events in categorized levels of on-clopidogrel platelet reactivity.

	<190 AU x min	191–468 AU x min	>468 AU x min
No bleeding	135 (55.6%)	84 (57.5%)	66 (69.5%)
BARC 1	17 (7.0%)	7 (4.8%)	5 (5.3%)
BARC 2	64 (26.3%)	41 (28.1%)	12 (12.6%)
BARC 3a	10 (4.1%)	6 (4.1%)	6 (6.3%)
BARC 3b	13 (5.3%)	6 (4.1%)	6 (6.3%)
BARC 3c	1 (0.4%)	1 (0.7%)	-
BARC 5a + b	3 (1.2%)	1 (0.7%)	-
All	243 (100%)	146 (100%)	95 (100%)

Severity of bleeding events categorized by cut-off values for high and low on-clopidogrel platelet reactivity.

**Table 4:** Logistic regression models.

	Univariate Model			Multivariate Model		
	Odds Ratio	95% CI	*p*-Value	Odds Ratio	95% CI	*p*-Value
On ASA platelet reactivity (AU x min)	1.0	0.999–1.001	0.806			
Low/normal/high on—Clopidogrel platelet reactivity	0.549	0.322–0.910	0.020	0.533	0.309–0.917	0.023
Oral anticoagulation	1.837	1.265–2.666	0.001	1.766	1.209–2.581	0.003
Age (years)	0.994	0.961–1.029	0.751			
Platelet count (10^3^/µL)	0.998	0.995–1.000	0.087	0.999	0.996–1.001	0.341
Hemoglobin (g/dL)	1.038	0.933–1.115	0.493			

Univariate and multivariate binary regression models to test if variables are predictive for bleeding events; ASA = acetylsalicylic acid.

## Data Availability

Not applicable.

## Supplementary Materials

The following supporting information can be downloaded at: https://www.mdpi.com/article/10.3390/jcm11102871/s1. Table S1: Results of multiple electrode impedance aggregometry.
