# Supplementary material for: Impact of On-Clopidogrel Platelet Reactivity on Incidence of Peri-Interventional Bleeding in Patients Undergoing Transcatheter Aortic Valve Implantation
Source: J Clin Med. 2022 May 19;11(10):2871. doi: 10.3390/jcm11102871 (PMC9148071; doi:10.3390/jcm11102871)
Supplement: Supplementary file 1 [file jcm-11-02871-s001.zip › jcm-1685736-supplementary.pdf]

Supplementary Table S1: Results of multiple electrode impedance aggregometry

	**On ASA platelet reactivity (AU x min)**			On clopidogrel platelet reactivity (AU x min)		
	Day 0	Day 1	Day 5	Day 0	Day 1	Day 5
All	99 [41 – 187]	39 [10 – 89]	120 [47 – 283]	179 [96 – 369]	95 [47 – 152]	119 [68 – 176]
No Bleeding	99 [41 – 184]	40 [14 – 89]	120 [48 – 277]	198 [103 – 408]	101 [48 – 166]	130 [85 – 191]
Bleeding	104 [41 – 191]	38 [3 – 97]	120 [40 – 294]	163 [73 – 305]	85 [47 – 132]	101 [54 – 159]
